# Marital and living status and biological ageing trajectories: a longitudinal cohort study with a 20-year follow-up

**DOI:** 10.1007/s10522-024-10171-1

**Published:** 2025-01-08

**Authors:** Weiyao Yin, Xia Li, Ruoqing Chen, Yiqiang Zhan, Juulia Jylhävä, Fang Fang, Sara Hägg

**Affiliations:** 1 Department of Medical Epidemiology and Biostatistics, Karolinska Institutet, Box 210, 171 77 Stockholm, Sweden; 2 Department of Obstetrics and Gynecology, West China Second University Hospital, Sichuan University, Chengdu, China; 3 School of Public Health and Emergency Management, Southern University of Science and Technology, Shenzhen, Guangdong China; 4 School of Public Health (Shenzhen), Sun Yat-Sen University, Shenzhen, China; 5 Institute of Environmental Medicine, Karolinska Institutet, Stockholm, Sweden; 6 Faculty of Medicine and Health Technology and Gerontology Research Center (GEREC), University of Tampere, Tampere, Finland; 7 Tampere Institute for Advanced Study, Tampere, Finland

**Keywords:** Biological ageing, Ageing trajectories, Frailty, Cognitive function, Living status, Marital status, Telomere, Cognition, Longitudinal

## Abstract

Biomarkers of ageing (BA) can predict health risks beyond chronological age, but little is known about how marital/living status affects longitudinal changes in BA. We examined the association between marital/living status and BA over time using the-Swedish-Adoption/Twin-Study-of-Aging (SATSA) cohort. Four BAs were analyzed: telomere length (TL) (638 individuals; 1603 measurements), DNAmAge (535 individuals; 1392 measurements), cognition (823 individuals; 3218 measurements), and frailty index (FI) (1828 individuals; 9502 measurements). Individuals were born between 1900 and 1948, and data on marital/living status, BAs, and covariates were collected through nine waves of questionnaires and in-person testing from 1986 to 2014. Mixed linear regression with random effects at twin-pair and individual levels were used to assess BA changes for constant marital/living status. Conditional generalized estimating equation assessed within-individual BA changes for varying marital/living status. Results showed that individuals who were consistently unmarried/non-cohabiting (β = 0.291, 95%CI = 0.189–0.393) or living alone (β = 0.203, 95%CI = 0.090–0.316) were more frail, and experienced accelerated frailty (p-for-interaction with age < 0.001 for marital status; p-for-interaction = 0.002 for living status) and cognitive decline (p-for-interaction < 0.001), compared to those married/cohabiting or living with someone Among individuals whose marital/living status changed, frailty was higher when living alone (β = 0.089, 95%CI = 0.017–0.162) and frailty accelerated when they became unmarried/non-cohabiting or were living alone (p-for-interaction < 0.001). Cognitive decline also accelerated when living alone (p-for-interaction = 0.020). No associations were observed for TL and DNAmAge. In conclusion, being unmarried/non-cohabiting or living alone from mid-to-old age is linked to accelerated cognitive decline and frailty. These findings highlight the potential importance of social support networks and living arrangements for healthy ageing.

**Supplementary Information:**

The online version contains supplementary material available at 10.1007/s10522-024-10171-1.

## Introduction

Ageing is characterized by a progressive loss of function throughout life, and an advanced age is generally associated with adverse health outcomes and excess risk of mortality (Jylhava et al. 2017). There are biomarkers and measures that are closely correlated with chronological age (CA), allowing for assessing molecular, functional and system-wide ageing process. Therefore, quantifications of these biomarkers of ageing (BA) can predict late-life health risks in individuals of the same CA, beyond CA alone (Jylhava et al. 2017; Li et al. 2020). Telomeres are nucleotide-protein complexes at the ends of chromosomes and shorten with every cell division (Blackburn et al. 2015). Short telomere length (TL) is associated with age-related consequences, e.g., cardiovascular diseases and overall mortality (Blackburn et al. 2015; Haycock et al. 2014). Epigenetic age is assessed by summarizing DNA methylation levels at several genomic loci, referred to as ‘epigenetic clocks’, applicable to multiple tissues and across the life course (Hannum et al. 2013; Horvath 2013). Changes in the levels of DNA methylation have been associated with age-related traits (Hannum et al. 2013; Horvath 2013). Cognitive changes start to manifest around retirement age, when declines in perception precede declines in other domains (Hägg and Jylhävä 2021). Frailty is a geriatric condition of reduced homeostasis and increased vulnerability to stressors. The Rockwood frailty index (FI) assesses medical conditions, physiological function, mental health and social wellbeing. Its sensitivity at the lower end of the scale makes the FI applicable to younger adults, providing a more comprehensive measure of the ageing process (Searle et al. 2008).

Previous studies have suggested that adverse life events, such as marital disruption in older age, may cause psychological distress and an accelerated ageing process (Chen et al. 2020; Fiorito et al. 2019; Mathur et al. 2016; Wolf et al. 2018). Shorter TL (Chen et al. 2020; Mainous et al. 2011; Whisman et al. 2016; Yen and Lung 2013), reduced cognitive performance (Chen et al. 2022; Costa-Cordella et al. 2021; Elovainio et al. 2018; Feng et al. 2014; Haghighi and Oremus 2023; Håkansson et al. 2009; Liao and Scholes 2017; Najar et al. 2021; Nakahori et al. 2021; Sommerlad et al. 2018; Sundström et al. 2016), and higher frailty (Chamberlain et al. 2016; Kojima et al. 2020; Pollack et al. 2017; Raymond et al. 2020; Young et al. 2016; Yu et al. 2018) have been reported in unmarried and non-cohabiting individuals. However, the results are inconsistent (Del Brutto et al. 2019; Hajek et al. 2016, 2020; Röhr et al. 2022; Scholes and Liao 2023; Trevisan et al. 2016, 2017, 2020; Vidarsdottir et al. 2014; Yu and Liu 2020). Thus far, studies on marital status and TL are cross-sectional in nature, lacking longitudinal approaches to capture changes of TL over time. Few studies on marital status and cognition are longitudinal (Costa-Cordella et al. 2021; Haghighi and Oremus 2023; Sommerlad et al. 2018), which is a limitation given the fluctuations in cognitive performance, especially in older age. Studies examining the effect of marital status on frailty often have short follow-up periods (Hajek et al. 2016, 2020; Pollack et al. 2017; Trevisan et al. 2016, 2017, 2020). Although psychological stress has been linked to increased epigenetic age, to our knowledge, no studies have investigated the association between marital/living status and longitudinal trajectories of epigenetic ageing (Fiorito et al. 2019; Wolf et al. 2018; Zannas et al. 2015). Furthermore, all previous studies have only assessed one single type of BA, limiting the ability to provide a multidimensional estimation of the ageing process or to directly compare different BA measures. Finally, little is currently known about the ageing trajectories before and after a change in marital/living status.

In this study, we examined the associations between marital/living status and multiple BAs in a longitudinal setting. We utilized nine waves of data collected through questionnaires and in-person testing (IPT) in the Swedish Adoption/Twin Study of Aging (SATSA) cohort, spanning a 20-year follow-up period (Finkel and Pedersen 2004). We employed a bottom-up approach, ranging from molecular BAs (TL and epigenetic age by Horvath) to functional BA (cognition) and system-wide BA (frailty).

## Materials and methods

### Study population

**Fig. 1:**
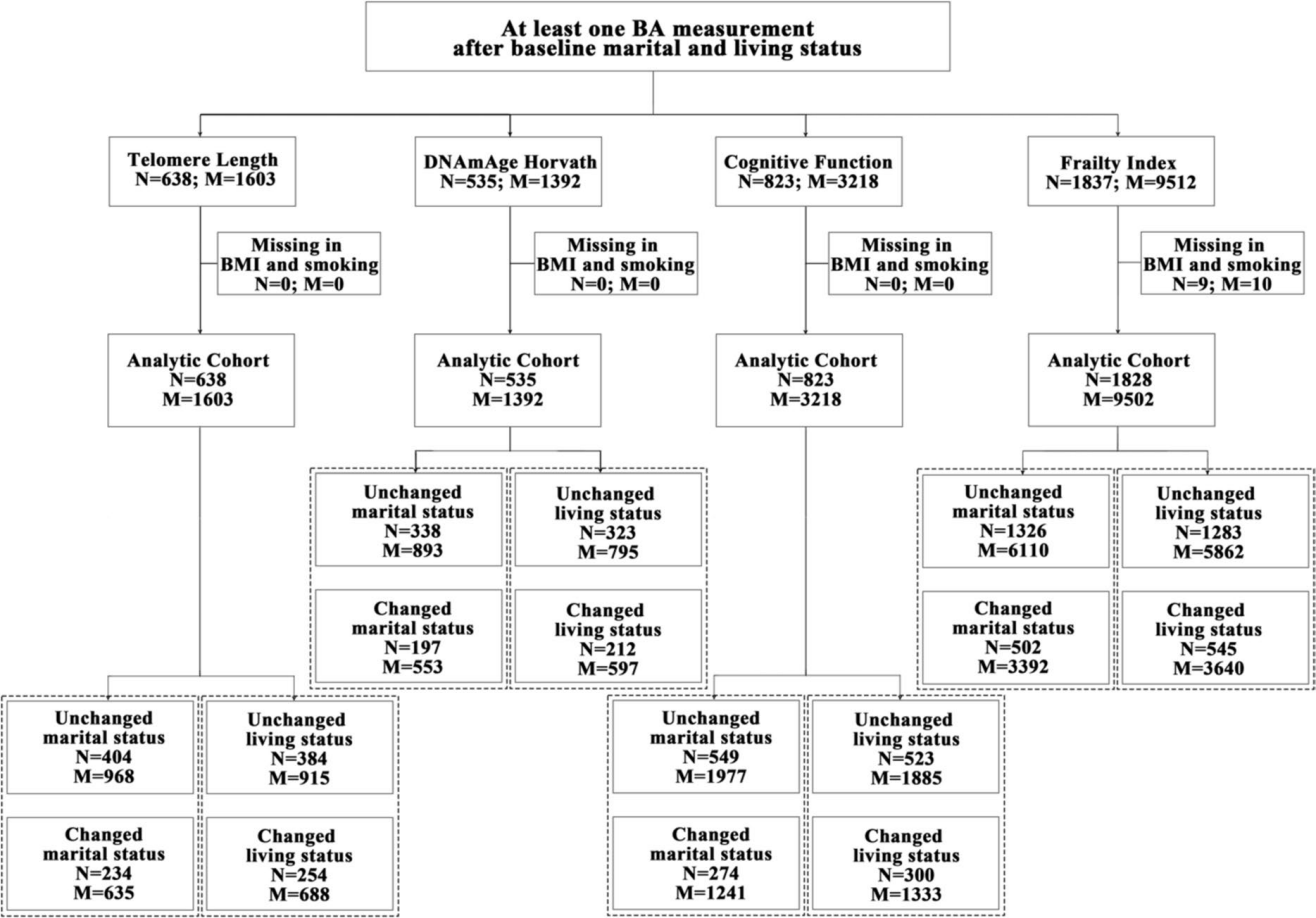
Flow chart. *BA* Biomarkers of ageing, *BMI* body mass index, *N* number of individuals, *M* number of measurements

SATSA is a population-based cohort including twin pairs reared together or apart in Sweden (Finkel and Pedersen 2004). By 2014, SATSA had completed up to nine waves of mail-out questionnaires (Q1–Q9) and IPTs (IPT1–IPT10, except for IPT4 which was a telephone interview) (eFigure 1). Information on physical and mental health status [e.g., body mass index (BMI)], lifestyle (e.g., smoking), socioeconomic status (e.g., marital status and educational attainment), and BA measurements was collected from one or multiple waves of questionnaires and IPTs. The samples for DNA extraction, DNA methylation and telomere length analyses were collected from different IPTs (from IPT3 to IPT 10), and were stored and processed together after the last wave of collection. In this way, the samples were randomized to avoid batch effects between different IPTs. The present study included 535 participants who had at least one record of marital/living status prior to the measurement of Horvath DNA methylation age measurement, 638 prior to TL measurement, 823 prior to cognitive measurement, and 1828 prior to frailty measurement from any of the questionnaires or IPTs (Fig. 1).

### Assessment of marital and living status

Marital status was assessed through the question ‘What is your current marital status?’ with four options: (1) married or cohabiting, (2) never married or cohabiting, (3) divorced or separated, and (4) widowed. In contrast to ‘married/cohabitating’, we combined married statuses of never married or cohabiting, divorced or separated, and widowed into one category ‘unmarried/non-cohabiting’. As a secondary measurement, living status was assessed by asking ‘What is your current living status?’ with three options: (1) living with partner, (2) living with children, relatives, friends and others, and (3) living alone. Based on the responses, we categorized living status into living with someone (partner and/or others) or living alone.

### Assessment of BAs

#### Telomere length (TL)

TL was measured from leukocyte DNA in peripheral blood. Standard quantitative polymerase chain reaction (qPCR) technique was applied for the measurement and a T/S ratio representing a relative length was calculated as described previously (Berglund et al. 2016; Cawthon 2002). In brief, telomere sequence copy gene (T) in each sample was compared to a single copy reference gene from β-hemoglobin (S). T/S-ratio values were further adjusted for batch effects and then rescaled back to the original distribution. The measurement methods of TL and other BAs are summarized in eTable 1.

#### Epigenetic clock

Epigenetic age was estimated based on ageing-related DNA methylation levels summarized from various genomic loci. We used the Horvath clock—a well-established DNA methylation-based age estimator across multiple tissue types and the entire lifespan (Hannum et al. 2013; Horvath 2013). In brief, genome-wide methylation levels of 353 CpGs were measured from whole blood leukocytes using the Illumina Infinium HumanMethylation450 BeadChip (Wang et al. 2018), from which Horvath DNAmAge (DNA methylation age) was calculated by the online DNA Methylation Age Calculator (Hannum et al. 2013; Horvath 2013).

#### Cognitive function

Cognitive performance was evaluated using a battery of in-person cognitive testing in four specific domains, i.e., verbal (crystallized), spatial (fluid), memory, and perceptual speed abilities. Scores on four cognitive domains were recorded to percentage correct of the total possible points for each respective test. A general cognitive ability score was derived through the principal component analysis of the tests (Reynolds et al. 2005). Component scoring coefficients from the first component extracted from the IPT1 (excluding persons with dementia) were used to construct a measure of cognitive functioning at IPT1 and subsequent IPTs using test scores standardized to the mean and SD of each test at IPT1. T-score scaling (M = 50, SD = 10) was then applied to the components (Li et al. 2020).

#### Frailty index (FI)

FI was constructed according to the Rockwood deficit accumulation model (Searle et al. 2008). In the model we included a set of 42 self-reported deficits that covered a wide range of health domains, including symptoms, diseases, activities in daily living, and mental health. The FI for each individual was assessed as a ratio with the number of deficits divided by the total number of deficits included (n = 42), leading to a value between 0 and 1 (Jiang et al. 2017). Details on the 42 items included in the FI are described in eTable 2.

### Assessment of covariates

BMI was calculated from weight and height (kg/m^2^), either measured during physical examinations or self-reported in questionnaires. Educational attainment (primary education, lower secondary or vocational education, upper secondary education, or tertiary education) and smoking status (non-smoker, ex-smoker, or current smoker) were obtained from self-reported questionnaires.

### Statistical analyses

Descriptive statistics was presented as mean (standard deviation, SD) or median (interquartile range, IQR, for FI) for continuous variables, and frequencies and proportions for categorical variables. Marital/living status at baseline was defined as the latest status reported before the first available BA measurement. Marital/living status during follow-up was obtained from the most recent information available prior to each BA measurement. BMI and smoking status were obtained from the questionnaire or IPT closest to each BA measurement. If data were missing, they were imputed from the next nearest available questionnaire or IPT. Birth year (1900–1948) was categorized into 10-year intervals. Since the distribution of FI was right skewed, it was transformed using a square-root to better fit a normal distribution (eFigure 2). To enable direct comparisons between BAs, we standardized all BAs to examine the association of marital/living status with per SD increase in each BA.

Longitudinal changes in BAs were assessed among people with constant marital/living status (Fig. 1). As in previous studies, CA was used as the time scale for the model due to its close relationship with BAs (Li et al. 2020). Average trajectories of BAs were presented as population-level means over CA, as a function of marital/living status, CA, sex, educational attainment, smoking, BMI and birth year, with random intercept at the twin-pair level accounting for shared genetics and non-genetic factors within a twin pair, as well as at the individual level accounting for individual-specific influences. We used the formula

$${BA}_{it} = {\beta }_{0}+ {\beta }_{1}*Marital/living status+ {\beta }_{2}*{CA}_{it}+ {\beta }_{3}*Sex+ {{\beta }_{4}*{Covariates}_{it}+\mu }_{0i}+ {\varepsilon }_{it}$$.

In this formula, i and t denote individual and measurement, respectively; β and µ indicate fixed and random effects, respectively; ε indicates random error. The longitudinal BA trajectories with different marital/living statuses were visualized by including an interaction term (marital/living status*CA). The estimated population-level BAs in each group were plotted using smooth lines and the individual-level BAs using connected lines.

All statistical tests were performed using two-sided 5% level of significance, corresponding to a two-sided 95% confidence interval (CI). We did not adjust p-values for multiplicity of statistical tests because the primary analyses involved a limited number of predefined comparisons addressing the primary research question. Specifically, these analyses focused on examining the relationship between two aspects of social status (married/cohabiting vs. unmarried/non-cohabiting, and living with someone vs. living alone) and BA levels. Statistical analyses were performed using R.3.6.0.

### Supplementary analyses

To provide a comprehensive view of the relationship across different temporal contexts, we conducted a cross-sectional analysis to assess the baseline association between marital/living status and BA, as well as a within-individual analysis to assess how changes in marital/living status over time affect BA. (1) Cross-sectional analysis: Among individuals with constant marital/living status, we examined the association between baseline marital/living status and the first measure of BAs. Mixed linear regression models with fixed effects for CA, sex, educational attainment, smoking, BMI and birth year, and random intercept at the twin-pair level, were applied. (2) Within-individual analysis: Among people with varying marital/living status, we examined the association between changes in martial/living status and within-individual changes in BAs using conditional generalized estimating equation (cGEE) model, adjusting for time-varying covariates including CA, smoking, and BMI. Additionally, the interaction between marital/living status and CA was examined. (3) To address sex-specific effects, we performed a formal test for interaction between marital/living status and sex.

## Results

### Baseline characteristics

After excluding individuals with missing data on BMI and smoking throughout the nine waves, the final analytic cohort included 638 individuals with 1603 measurements of TL, 535 individuals with 1392 measurements of Horvath DNAmAge, 823 individuals with 3218 measurements of cognitive function, and 1828 individuals with 9502 measurements of FI **(**Fig. 1). The average baseline value was 0.70 for TL (T/S ratio), 61.2 years for Horvath DNAmAge, 51.8 for cognitive function, and 0.08 for FI. The mean age at first available BA measurement was 68.8, 68.3, 63.8 and 62.0 years for the participants in the analyses of TL, Horvath DNAmAge, cognitive function, and FI, respectively. The difference in mean age was partly due to the availability of data in IPTs (TL and DNAmAge available from IPT3, cognitive function from IPT1, and FI from Q2). Characteristics at baseline across four BA groups are summarized in Table 1.

**Table 1:** Characteristics in individuals with information on at least one marital and living status before BA measurements

	Telomere length (T/S ratio)	DNAmAge Horvath	Cognitive function	Frailty index
		(years)		
Number of participants	638	535	823	1828
Total measurements	1603	1392	3218	9502
Individual’s measurements (median, IQR)	2 (1–4)	2 (1–4)	3 (2–6)	4 (2–7)
Follow-up years (mean ± SD)	19.4 (7.1)	19.7 (7.1)	15.5 (9.5)	15.8 (10.3)
*Baseline characteristics*				
Marital and living status				
Married/cohabiting (Yes; N, %)	427 (66.9)	365 (68.2)	609 (74.0)	1212 (66.3)
Living with someone (Yes; N, %)	449 (70.4)	387 (72.3)	648 (78.7)	1347 (73.7)
Age at baseline martial and living status (yrs, mean ± SD)	57.1 (10.3)	56.7 (10.3)	59.1 (10.6)	59.0 (13.6)
BA measurements				
BA level: 1st (median, IQR)	0.70 (0.62–0.81)	61.19 (55.32–68.14)	51.84 (44.81–58.93)	0.08 (0.04–0.14)
Age at first BA measurement (yrs, mean ± SD)	68.8 (9.6)	68.3 (9.5)	63.8 (8.3)	62.0 (13.7)
Men (N, %)	266 (41.7)	221 (41.3)	331 (40.2)	764 (41.8)
BMI (kg/m^2^, mean ± SD)	26.3 (4.1)	26.4 (4.2)	25.7 (4.0)	24.7 (3.7)
Educational attainment (N, %)				
Elementary school	348 (54.5)	284 (53.1)	470 (57.1)	959 (52.5)
O-level or vocational school or folk school	178 (27.9)	156 (29.2)	221 (26.9)	443 (24.2)
Gymnasium (A-level)	46 (7.2)	37 (6.9)	53 (6.4)	192 (6.8)
University or higher	42 (6.6)	39 (7.3)	53 (6.4)	109 (6.0)
Unknown	24 (3.8)	19 (3.6)	26 (3.2)	125 (10.5)
Smoking status (N, %)				
Never	500 (78.4)	421 (78.7)	603 (73.3)	1297 (71.0)
Former	25 (3.9)	21 (3.9)	58 (7.1)	105 (5.74)
Current	113 (17.7)	93 (17.4)	162 (19.7)	426 (23.3)

The median of frailty index presented, as it is rightly skewed

*SD* standard deviations, *BA* biomarkers of ageing, *BMI* body mass index, *IQR* interquartile range

### Longitudinal trajectories of BAs in individuals with constant marital/living status

**Fig. 2:**
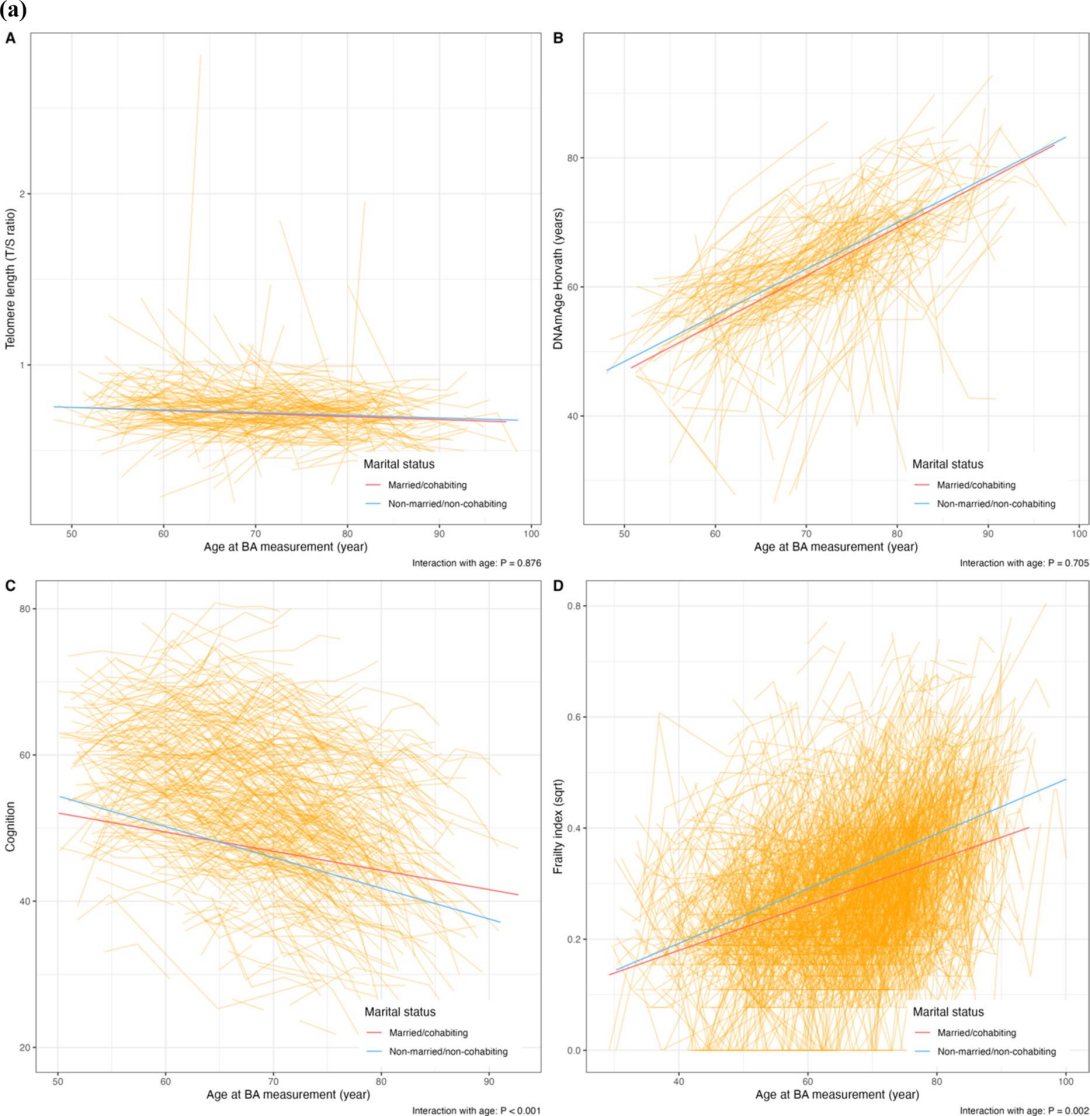

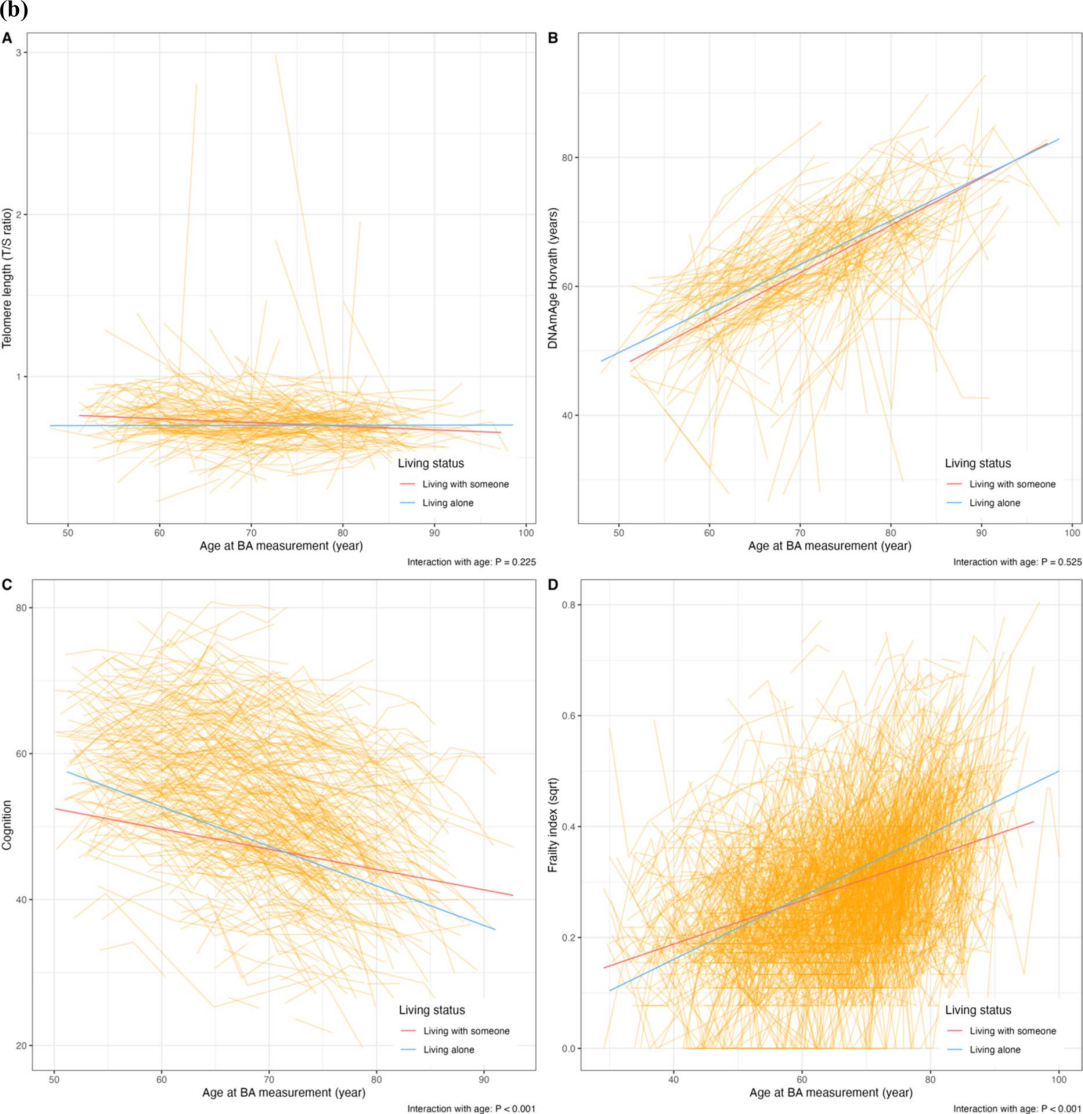
Longitudinal trajectories of BA in people with constant marital and living status. **a** Longitudinal trajectories of BA by marital status. **b** Longitudinal trajectories of BA by living status. *Note* Average changes of BA with chronological age were denoted by smooth lines, with red representing individuals who were married/cohabiting (**a**) or living with someone (**b**), and blue for being unmarried/non-cohabiting (**a**) or living alone (**b**). Individual BA measurements were presented as orange-connected lines. Mixed linear regression models were used to estimate longitudinal changes of BA, with fixed effects at chronological age, sex, education attainment, smoking status, BMI and calendar year of birth, including an interaction term between marital status and chronological age or living status and chronological age, and with random effects at the twin-pair level and the individual level

Individuals who were unmarried/non-cohabiting had a higher FI (fully-adjusted β 0.291, 95%CI 0.189–0.393) than those who were married/cohabiting; similarly, a higher FI was observed among individuals living alone than individuals living with someone (fully-adjusted β 0.203, 95%CI 0.090–0.316) (Table 2). Cognitive function was lower in unmarried/non-cohabiting individuals than that of married/cohabiting individuals when CA and sex were adjusted for; however, the difference became non-statistically significant after further adjusting for BMI and smoking. No association was observed between living status and cognitive function, nor between martial/living status and molecular BAs. The longitudinal trajectories of BAs over CA by marital/living status are visualized in Fig. 2a, b. There were statistically significant interactions between marital/living status and CA in relation to cognitive function and FI, suggesting that being unmarried/non-cohabiting or living alone accelerated cognitive decline and frailty over time.

**Table 2:** The association between marital and living status and repeated BA measurements among people with constant marital and living status

BA measurements	Total measurements	Model 1	Model 2
		β and 95%CI	β and 95%CI
*Telomere length (T/S ratio)*			
Marital status			
Married/cohabiting	693	Reference	Reference
Unmarried/non-cohabiting	275	0.022 (− 0.157, 0.200)	0.031 (− 0.153, 0.215)
Living status			
Living with someone	724	Reference	Reference
Living alone	191	− 0.021 (− 0.236, 0.195)	− 0.021 (− 0.244, 0.203)
*DNAmAge Horvath (years)*			
Marital status			
Married/cohabiting	618	Reference	Reference
Unmarried/non-cohabiting	221	0.059 (− 0.104, 0.222)	0.095 (− 0.070, 0.260)
Living status			
Living with someone	649	Reference	Reference
Living alone	146	0.038 (− 0.153, 0.228)	0.094 (− 0.102, 0.287)
*Cognitive function*			
Marital status			
Married/cohabiting	1444	Reference	Reference
Unmarried/non-cohabiting	533	− 0.232 (− 0.401, − 0.066)	− 0.096 (− 0.247, 0.054)
Living status			
Living with someone	1516	Reference	Reference
Living alone	369	− 0.156 (− 0.344, 0.031)	− 0.016 (− 0.189, 0.156)
*Frailty index (square root)*			
Marital status			
Married/cohabiting	4321	Reference	Reference
Unmarried/non-cohabiting	1789	0.310 (0.211, 0.409)	0.291 (0.189, 0.393)
Living status			
Living with someone	4641	Reference	Reference
Living alone	1221	0.233 (0.123, 0.343)	0.203 (0.090, 0.316)

Mixed models were used to estimate the association between baseline marital status and repeated BA measurement in one-SD increase, with fixed effects for chronological age and sex in Model 1, and additionally for educational attainment, smoking status, BMI and birth year in 10-year interval in Model 2, and with random intercepts at the twin-pair level and the individual level

*BA* biomarkers of ageing, *CI* confidence interval

### Supplementary analyses

(1) Cross-sectional analysis: Among individuals with constant marital/living status, compared to individuals who were married/cohabitating, unmarried/non-cohabiting individuals had a higher level of FI (fully-adjusted β 0.285, 95%CI 0.169–0.399). A higher FI was also found in individuals living alone than individuals living with someone (fully-adjusted β 0.183, 95%CI 0.056–0.309). There was no evidence to support associations between marital/living status and TL, DNAmAge and cognitive function (eTable 3). (2) Within-individual analysis: Among individuals who changed marital/living status, FI increased when the individual lived alone, compared to when they lived with someone (fully-adjusted model: β 0.089, 95%CI 0.017–0.162) (Table 3). FI also increased when the individual was unmarried/non-cohabiting, compared to when they were married/cohabitating, after adjusting for CA and sex (β 0.085, 95%CI 0.014–0.156). However, the association became non-statistically significant with further adjustment for BMI and smoking. Individuals had an accelerated rate of frailty (interaction between marital/living status and CA, p < 0.001) and cognitive decline (interaction between living status and CA, p = 0.02) when in the status of unmarried/non-cohabitating or living alone, compared to in the status of married/cohabitating or living with someone, respectively. There was no sex difference in the associations between marital/living status and all BA measures (eTable 4).

**Table 3:** Within-individual BA measurements in people with varying marital and living status

BA measurements	Total measurements	Model 1		Model 2	
		β and 95%CI	*P* for interaction	β and 95%CI	*P* for interaction
*Telomere length (T/S ratio)*					
Marital status	635				
Married/cohabiting	354	Reference	–	Reference	–
Unmarried/non-cohabiting	281	− 0.249 (− 0.548, 0.049)		− 0.254 (− 0.551, 0.044)	
Interaction with age		− 0.002 (− 0.019, 0.015)	0.790	− 0.002 (− 0.019, 0.015)	0.835
Living status	688				
Living with someone	366	Reference	–	Reference	–
Living alone	322	0.027 (− 0.209, 0.263)		0.031 (− 0.201, 0.264)	
Interaction with age		− 0.007 (− 0.026, 0.013)	0.509	− 0.007 (− 0.026, 0.013)	0.497
*DNAmAge Horvath (years)*					
Marital status	553				
Married/cohabiting	308	Reference	–		
Unmarried/non-cohabiting	245	− 0.012 (− 0.182, 0.157)		− 0.004 (− 0.174, 0.166)	
Interaction with age		− 0.004 (− 0.022, 0.013)	0.618	− 0.005 (− 0.023, 0.013)	0.580
Living status	597				
Living with someone	323				
Living alone	274	− 0.058 (− 0.217, 0.100)		− 0.058 (− 0.215, 0.099)	
Interaction with age		− 0.005 (− 0.023, 0.012)	0.553	− 0.005 (− 0.022, 0.012)	0.600
*Cognitive function*					
Marital status	1241				
Married/cohabiting	807	Reference	–	Reference	–
Unmarried/non-cohabiting	434	0.014 (− 0.052, 0.080)		0.008 (− 0.057, 0.074)	
Interaction with age		− 0.008 (− 0.016, 0.000)	0.04	− 0.007 (− 0.015, 0.000)	0.058
Living status	1333				
Living with someone	857	Reference	–	Reference	–
Living alone	476	0.050 (− 0.021, 0.122)		0.049 (− 0.021, 0.119)	
Interaction with age		− 0.009 (− 0.017, − 0.002)	0.016	− 0.009 (− 0.017, − 0.001)	0.020
*Frailty index (square root)*					
Marital status	3392				
Married/cohabiting	1848	Reference	–	Reference	–
Unmarried/non-cohabiting	1544	0.085 (0.014, 0.156)		0.061 (− 0.008, 0.130)	
Interaction with age		0.011 (0.006, 0.016)	< 0.001	0.009 (0.005, 0.014)	< 0.001
Living status	3640				
Living with someone	1978	Reference	–	Reference	
Living alone	1662	0.107 (0.032, 0.182)		0.089 (0.017, 0.162)	
Interaction with age		0.011 (0.006, 0.016)	< 0.001	0.010 (0.005, 0.014)	< 0.001

The conditional generalized estimating equation (cGEE) model was used for within-individual analysis, adjusted for CA in Model 1 and additionally for smoking and BMI in Model 2 (sex, birth year and educational attainment did not change within the same individual therefore were not included in the cGEE models), conditioning on individuals. The interaction between marital status and CA as well as living status and CA were further tested by interaction terms

*BA* biomarkers of ageing, *CI* confidence interval

## Discussion

Through studying both molecular/cellular processes and system-wide functioning, we examined the impact of marital/living status on four biomarkers of ageing in a longitudinal cohort with a 20-year follow-up. We found that individuals who were constantly unmarried/non-cohabiting or living alone tended to be frailer, and experienced accelerated frailty and cognitive decline over time. For those with changed marital/living status, their frailty levels were higher when living alone, and frailty and cognitive decline progressed more rapidly when they were unmarried/non-cohabitating or living alone, compared to when they were married/cohabiting or living with someone, respectively. These findings are mostly noted for cognitive decline and frailty—two organism-level ageing indicators. We did not find evidence for influences of marital/living status on TL or epigenetic age—biomarkers of ageing process at the cellular level.

The adverse impact of life stressors has been shown to influence the ageing process (Fiorito et al. 2019; Mathur et al. 2016; Wolf et al. 2018). Losing a spouse or ending a marriage, especially in middle or old age, could be highly stressful (Lorenz et al. 2006). Chronic stress has been linked to multiple interconnected biological mechanisms that accelerate ageing, including increased burden of oxidative stress (Mainous et al. 2011), higher inflammatory response (O'Donovan et al. 2011; Segerstrom and Miller 2004), changes in DNA methylation patterns (Zannas et al. 2015) and impaired cognitive function (Lupien et al. 2009). Living alone in later life often implies social isolation and limited social interaction, which can contribute to a range of physical and mental health challenges, including chronic stress, depression, cognitive impairment, somatic diseases, and increased mortality (Costa-Cordella et al. 2021). In the present study, advanced functional ageing, such as frailty and cognitive decline, but not cellular changes, such as TL and epigenetic age, was linked to negative marital/living status. Compared to molecular/cellular changes, functional/system-wide assessments provide a more holistic perspective on an individual’s overall resilience and well-being. Notably, these does not imply that the organism decline occurs independently of cellular decline. In fact, cellular ageing typically precedes functional ageing (Mak et al. 2024). While the frailty index can reflect a general, whole-organism level decline, it is highly likely that cellular decline is also occurring, but may not be detectable with the methods employed in the present study. The underlying mechanisms warrant further investigation.

To the best of our knowledge, this is the first study assessing the association between marital/living status and longitudinal changes of TL. Previous cross-sectional studies have reported a shorter TL among people living alone (Chen et al. 2020), unmarried individuals (Mainous et al. 2011; Yen and Lung 2013), and after marital disruption (Whisman et al. 2016). Another cross-sectional study found no association between marital quality and TL among women, while it revealed that positive and negative marital quality affected men’s TL differently (Yu and Liu 2020). In the present study, marital/living status was not associated with TL. Although psychological stress has been linked to changes in DNA methylation (Fiorito et al. 2019; Wolf et al. 2018; Zannas et al. 2015), little is known regarding the impact of marital/living status on the epigenetic ageing markers. The result of our study did not support an accelerated Horvath clock among people who were unmarried/non-cohabiting or living alone. However, given the complex nature of cellular ageing processes (Yu and Liu 2020), more research is needed to validate these findings, particularly utilizing the latest updated clocks and biomolecular techniques to separate beneficial adaptions and pathological changes in epigenetic age (Ying et al. 2024).

Previous studies generally suggest marital partner as a protective buffer against cognitive decline or dementia (Costa-Cordella et al. 2021; Haghighi and Oremus 2023; Sommerlad et al. 2018), which is in line with our finding of an accelerated rate of cognitive decline in individuals who were unmarried/non-cohabiting or living alone. Very few of the existing studies in the field are of longitudinal design (Elovainio et al. 2018; Liao and Scholes 2017; Scholes and Liao 2023). Two studies have so far separately examined cognitive trajectory in women and men. One study found that higher spousal strain was associated with faster verbal memory decline in men (Scholes and Liao 2023) while another reported that having a spouse was associated with a slower cognitive decline in men (Liao and Scholes 2017). In our study, being unmarried/non-cohabiting or living alone was consistently associated with an unfavorable cognitive trajectory, without showing evidence of sex differences.

A meta-analysis based on 35 cross-sectional studies reported that unmarried individuals were nearly twice as likely to be physically frail (Kojima et al. 2020). Longitudinal studies on the impact of marital/living status on frailty have shown inconsistent results. Five studies associated non-cohabitation or living alone with higher frailty (Chamberlain et al. 2016; Pollack et al. 2017; Raymond et al. 2020; Young et al. 2016; Yu et al. 2018), whereas others reported no effect (Hajek et al. 2016, 2020), decreased frailty (Trevisan et al. 2017) or mixed results depending on age and sex (Trevisan et al. 2016, 2020). Most of these studies had limited frailty measurements and relatively short follow-up periods (Hajek et al. 2016, 2020; Pollack et al. 2017; Trevisan et al. 2016, 2017, 2020). Our previous study (Raymond et al. 2020) also showed a positive association between a marital status of non-cohabitation or living alone and increased frailty. In the present study, we extended our previous study to a longitudinal setting with repeated measurements of frailty, and found a higher level of frailty level and accelerated frailty trajectory in relation to being unmarried/non-cohabiting or living alone, using being married/cohabiting or living with someone as the reference.

Interestingly, in the present study, the association between being unmarried/non-cohabiting and a lower level of cognitive function disappeared after further adjusting for BMI and smoking. Similarly, within-individual differences in FI between being married/cohabiting and being unmarried/non-cohabiting also disappeared after adjusting for BMI and smoking. This indicates that BMI and smoking may partly explain the association between marital/living status and these BAs, either as confounders or mediators, consistent with previous studies showing associations between BMI, smoking, lower cognitive performance, and higher frailty (Jayanama et al. 2022; Lv et al. 2023; Zhang et al. 2022).

The strengths of our study include the longitudinal design with a long follow-up, and the use of diverse BAs ranging from the molecular/cellular markers to system-wide functioning. However, several limitations should be acknowledged. The study population mainly consisted of individuals with marital status records from middle to older age, a period characterized by increased frailty and reduced resilience, which may limit generalizability of the findings to younger populations. Furthermore, the observational nature of the study means that residual confounding from unmeasured factors, such as other life stressors, cannot be ruled out. As such, caution is advised in drawing causal conclusions, and further research is needed to elucidate the mechanisms linking marital and living conditions to ageing.

## Conclusion

Individuals who were unmarried/non-cohabiting or living alone from middle to old age appeared to experience accelerated cognitive decline and frailty. The findings highlight the potential importance of social support networks and living arrangements in relation to healthy ageing.

## Supplementary Information

Below is the link to the electronic supplementary material.

Supplementary file1 (PDF 363 KB)

## Data Availability

Data from SATSA are not publicly available due to ethical reasons. Data are available to qualified investigators upon request.

## References

[CR1] Berglund K, Reynolds CA, Ploner A, Gerritsen L, Hovatta I, Pedersen NL, Hagg S (2016) Longitudinal decline of leukocyte telomere length in old age and the association with sex and genetic risk. Aging-Us 8(7):1398–1415. 10.18632/aging.100995 10.18632/aging.100995 PMC4993338 27391763

[CR2] Blackburn EH, Epel ES, Lin J (2015) Human telomere biology: a contributory and interactive factor in aging, disease risks, and protection. Science 350(6265):1193–1198. 10.1126/science.aab3389 26785477 10.1126/science.aab3389

[CR3] Cawthon RM (2002) Telomere measurement by quantitative PCR. Nucleic Acids Res 30(10):e47. 10.1093/nar/30.10.e47 12000852 10.1093/nar/30.10.e47 PMC115301

[CR4] Chamberlain AM, St Sauver JL, Jacobson DJ, Manemann SM, Fan C, Roger VL, Yawn BP, Finney Rutten LJ (2016) Social and behavioural factors associated with frailty trajectories in a population-based cohort of older adults. BMJ Open 6(5):e011410. 10.1136/bmjopen-2016-011410 27235302 10.1136/bmjopen-2016-011410 PMC4885446

[CR5] Chen R, Zhan Y, Pedersen N, Fall K, Valdimarsdóttir UA, Hägg S, Fang F (2020) Marital status, telomere length and cardiovascular disease risk in a Swedish prospective cohort. Heart 106(4):267–272. 10.1136/heartjnl-2019-315629 31727634 10.1136/heartjnl-2019-315629

[CR6] Chen ZC, Wu H, Wang XD, Zeng Y, Huang G, Lv Y, Niu J, Meng X, Cai P, Shen L, Gang B, You Y, Lv Y, Ren Z, Shi Z, Ji Y (2022) Association between marital status and cognitive impairment based on a cross-sectional study in China. Int J Geriatr Psychiatry. 10.1002/gps.5649 34729814 10.1002/gps.5649

[CR7] Costa-Cordella S, Arevalo-Romero C, Parada FJ, Rossi A (2021) Social support and cognition: a systematic review. Front Psychol 12:637060. 10.3389/fpsyg.2021.637060 33708164 10.3389/fpsyg.2021.637060 PMC7941073

[CR8] Del Brutto OH, Mera RM, Zambrano M (2019) Cognitive decline is not influenced by the marital status or living arrangements in community-dwelling adults living in a rural setting. A population-based prospective cohort study. J Clin Neurosci 69:109–113. 10.1016/j.jocn.2019.08.019 31466904 10.1016/j.jocn.2019.08.019

[CR9] Elovainio M, Sommerlad A, Hakulinen C, Pulkki-Råback L, Virtanen M, Kivimäki M, Singh-Manoux A (2018) Structural social relations and cognitive ageing trajectories: evidence from the Whitehall II cohort study. Int J Epidemiol 47(3):701–708. 10.1093/ije/dyx209 29121238 10.1093/ije/dyx209 PMC6005021

[CR10] Feng L, Ng XT, Yap P, Li J, Lee TS, Håkansson K, Kua EH, Ng TP (2014) Marital status and cognitive impairment among community-dwelling Chinese older adults: the role of gender and social engagement. Dement Geriatr Cogn Dis Extra 4(3):375–384. 10.1159/000358584 25473404 10.1159/000358584 PMC4241637

[CR11] Finkel D, Pedersen NL (2004) Processing speed and longitudinal trajectories of change for cognitive abilities: the Swedish adoption/twin study of aging. Aging Neuropsychol Cogn 11(2–3):325–345. 10.1080/13825580490511152

[CR12] Fiorito G, McCrory C, Robinson O, Carmeli C, Rosales CO, Zhang Y, Colicino E, Dugué PA, Artaud F, McKay GJ, Jeong A, Mishra PP, Nøst TH, Krogh V, Panico S, Sacerdote C, Tumino R, Palli D, Matullo G, Guarrera S, Gandini M, Bochud M, Dermitzakis E, Muka T, Schwartz J, Vokonas PS, Just A, Hodge AM, Giles GG, Southey MC, Hurme MA, Young I, McKnight AJ, Kunze S, Waldenberger M, Peters A, Schwettmann L, Lund E, Baccarelli A, Milne RL, Kenny RA, Elbaz A, Brenner H, Kee F, Voortman T, Probst-Hensch N, Lehtimäki T, Elliot P, Stringhini S, Vineis P, Polidoro S (2019) Socioeconomic position, lifestyle habits and biomarkers of epigenetic aging: a multi-cohort analysis. Aging (Albany NY) 11(7):2045–2070. 10.18632/aging.101900 31009935 10.18632/aging.101900 PMC6503871

[CR13] Hägg S, Jylhävä J (2021) Sex differences in biological aging with a focus on human studies. Elife. 10.7554/eLife.63425 33982659 10.7554/eLife.63425 PMC8118651

[CR14] Haghighi P, Oremus M (2023) Examining the association between functional social support, marital status, and memory: a systematic review. BMC Geriatr 23(1):290. 10.1186/s12877-023-03982-3 37173618 10.1186/s12877-023-03982-3 PMC10182629

[CR15] Hajek A, Brettschneider C, Posselt T, Lange C, Mamone S, Wiese B, Weyerer S, Werle J, Fuchs A, Pentzek M, Stein J, Luck T, Bickel H, Mösch E, Heser K, Jessen F, Maier W, Scherer M, Riedel-Heller SG, König HH (2016) Predictors of frailty in old age—results of a longitudinal study. J Nutr Health Aging 20(9):952–957. 10.1007/s12603-015-0634-527791226 10.1007/s12603-015-0634-5

[CR16] Hajek A, Brettschneider C, Röhr S, Gühne U, van der Leeden C, Lühmann D, Mamone S, Wiese B, Weyerer S, Werle J, Fuchs A, Pentzek M, Weeg D, Mösch E, Heser K, Wagner M, Maier W, Riedel-Heller SG, Scherer M, König HH (2020) Which factors contribute to frailty among the oldest old? Results of the multicentre prospective AgeCoDe and AgeQualiDe study. Gerontology 66(5):460–466. 10.1159/000508723 32634802 10.1159/000508723

[CR17] Håkansson K, Rovio S, Helkala EL, Vilska AR, Winblad B, Soininen H, Nissinen A, Mohammed AH, Kivipelto M (2009) Association between mid-life marital status and cognitive function in later life: population based cohort study. BMJ 339:b2462. 10.1136/bmj.b2462 19574312 10.1136/bmj.b2462 PMC2714683

[CR18] Hannum G, Guinney J, Zhao L, Zhang L, Hughes G, Sadda S, Klotzle B, Bibikova M, Fan JB, Gao Y, Deconde R, Chen M, Rajapakse I, Friend S, Ideker T, Zhang K (2013) Genome-wide methylation profiles reveal quantitative views of human aging rates. Mol Cell 49(2):359–367. 10.1016/j.molcel.2012.10.016 23177740 10.1016/j.molcel.2012.10.016 PMC3780611

[CR19] Haycock PC, Heydon EE, Kaptoge S, Butterworth AS, Thompson A, Willeit P (2014) Leucocyte telomere length and risk of cardiovascular disease: systematic review and meta-analysis. BMJ 349:g4227. 10.1136/bmj.g4227 25006006 10.1136/bmj.g4227 PMC4086028

[CR20] Horvath S (2013) DNA methylation age of human tissues and cell types. Genome Biol 14(10):R115. 10.1186/gb-2013-14-10-r115 24138928 10.1186/gb-2013-14-10-r115 PMC4015143

[CR21] Jayanama K, Theou O, Godin J, Mayo A, Cahill L, Rockwood K (2022) Relationship of body mass index with frailty and all-cause mortality among middle-aged and older adults. BMC Med 20(1):404. 10.1186/s12916-022-02596-7 36280863 10.1186/s12916-022-02596-7 PMC9594976

[CR22] Jiang M, Foebel AD, Kuja-Halkola R, Karlsson I, Pedersen NL, Hagg S, Jylhava J (2017) Frailty index as a predictor of all-cause and cause-specific mortality in a Swedish population-based cohort. Aging (Albany NY) 9(12):2629–2646. 10.18632/aging.101352 29273703 10.18632/aging.101352 PMC5764396

[CR23] Jylhava J, Pedersen NL, Hagg S (2017) Biological age predictors. EBioMedicine. 10.1016/j.ebiom.2017.03.046 28396265 10.1016/j.ebiom.2017.03.046 PMC5514388

[CR24] Kojima G, Walters K, Iliffe S, Taniguchi Y, Tamiya N (2020) Marital status and risk of physical frailty: a systematic review and meta-analysis. J Am Med Dir Assoc 21(3):322–330. 10.1016/j.jamda.2019.09.017 31740150 10.1016/j.jamda.2019.09.017

[CR25] Li X, Ploner A, Wang Y, Magnusson PK, Reynolds C, Finkel D, Pedersen NL, Jylhävä J, Hägg S (2020) Longitudinal trajectories, correlations and mortality associations of nine biological ages across 20-years follow-up. Elife. 10.7554/eLife.51507 32041686 10.7554/eLife.51507 PMC7012595

[CR26] Liao J, Scholes S (2017) Association of social support and cognitive aging modified by sex and relationship type: a prospective investigation in the English longitudinal study of ageing. Am J Epidemiol 186(7):787–795. 10.1093/aje/kwx142 28520853 10.1093/aje/kwx142 PMC5860624

[CR27] Lorenz FO, Wickrama KA, Conger RD, Elder GH Jr (2006) The short-term and decade-long effects of divorce on women’s midlife health. J Health Soc Behav 47(2):111–125. 10.1177/002214650604700202 16821506 10.1177/002214650604700202

[CR28] Lupien SJ, McEwen BS, Gunnar MR, Heim C (2009) Effects of stress throughout the lifespan on the brain, behaviour and cognition. Nat Rev Neurosci 10(6):434–445. 10.1038/nrn2639 19401723 10.1038/nrn2639

[CR29] Lv J, Wu L, Sun S, Yu H, Shen Z, Xu J, Zhu J, Chen D, Jiang M (2023) Smoking, alcohol consumption, and frailty: a Mendelian randomization study. Front Genet 14:1092410. 10.3389/fgene.2023.1092410 36816044 10.3389/fgene.2023.1092410 PMC9935614

[CR30] Mainous AG 3rd, Everett CJ, Diaz VA, Baker R, Mangino M, Codd V, Samani NJ (2011) Leukocyte telomere length and marital status among middle-aged adults. Age Ageing 40(1):73–78. 10.1093/ageing/afq118 20817935 10.1093/ageing/afq118 PMC3000178

[CR31] Mak JKL, Karlsson IK, Tang B, Wang Y, Pedersen NL, Hägg S, Jylhävä J, Reynolds CA (2024) Temporal dynamics of epigenetic aging and frailty from midlife to old age. J Gerontol A Biol Sci Med Sci. 10.1093/gerona/glad251 37889476 10.1093/gerona/glad251 PMC11421301

[CR32] Mathur MB, Epel E, Kind S, Desai M, Parks CG, Sandler DP, Khazeni N (2016) Perceived stress and telomere length: a systematic review, meta-analysis, and methodologic considerations for advancing the field. Brain Behav Immun 54:158–169. 10.1016/j.bbi.2016.02.002 26853993 10.1016/j.bbi.2016.02.002 PMC5590630

[CR33] Najar J, Aakre JA, Vassilaki M, Wetterberg H, Rydén L, Zettergren A, Skoog I, Jack CR, Knopman DS, Petersen RC, Kern S, Mielke MM (2021) Sex difference in the relation between marital status and dementia risk in two population-based cohorts. J Alzheimers Dis 83(3):1269–1279. 10.3233/jad-210246 34420952 10.3233/JAD-210246 PMC8490335

[CR34] Nakahori N, Sekine M, Yamada M, Tatsuse T, Kido H, Suzuki M (2021) Association between marital status and cognitive function in Japan: results from the Toyama Dementia Survey. Psychogeriatrics 21(4):627–635. 10.1111/psyg.12724 34034362 10.1111/psyg.12724

[CR35] O’Donovan A, Pantell MS, Puterman E, Dhabhar FS, Blackburn EH, Yaffe K, Cawthon RM, Opresko PL, Hsueh WC, Satterfield S, Newman AB, Ayonayon HN, Rubin SM, Harris TB, Epel ES (2011) Cumulative inflammatory load is associated with short leukocyte telomere length in the Health, Aging and Body Composition Study. PLoS ONE 6(5):e19687. 10.1371/journal.pone.0019687 21602933 10.1371/journal.pone.0019687 PMC3094351

[CR36] Pollack LR, Litwack-Harrison S, Cawthon PM, Ensrud K, Lane NE, Barrett-Connor E, Dam TT (2017) Patterns and predictors of frailty transitions in older men: the osteoporotic fractures in men study. J Am Geriatr Soc 65(11):2473–2479. 10.1111/jgs.15003 28873220 10.1111/jgs.15003 PMC5681371

[CR37] Raymond E, Reynolds CA, Dahl Aslan AK, Finkel D, Ericsson M, Hägg S, Pedersen NL, Jylhävä J (2020) Drivers of frailty from adulthood into old age: results from a 27-year longitudinal population-based study in Sweden. J Gerontol A Biol Sci Med Sci 75(10):1943–1950. 10.1093/gerona/glaa106 32348465 10.1093/gerona/glaa106 PMC7518563

[CR38] Reynolds CA, Finkel D, McArdle JJ, Gatz M, Berg S, Pedersen NL (2005) Quantitative genetic analysis of latent growth curve models of cognitive abilities in adulthood. Dev Psychol 41(1):3–16. 10.1037/0012-1649.41.1.3 15656733 10.1037/0012-1649.41.1.3

[CR39] Röhr S, Pabst A, Baber R, Engel C, Glaesmer H, Hinz A, Schroeter ML, Witte AV, Zeynalova S, Villringer A, Löffler M, Riedel-Heller SG (2022) Social determinants and lifestyle factors for brain health: implications for risk reduction of cognitive decline and dementia. Sci Rep 12(1):12965. 10.1038/s41598-022-16771-6 35902604 10.1038/s41598-022-16771-6 PMC9334303

[CR40] Scholes S, Liao J (2023) Social support, social strain and declines in verbal memory: sex-specific associations based on 16-year follow-up of the English longitudinal study of ageing cohort. Aging Ment Health 27(4):780–788. 10.1080/13607863.2022.2089628 35735097 10.1080/13607863.2022.2089628

[CR41] Searle SD, Mitnitski A, Gahbauer EA, Gill TM, Rockwood K (2008) A standard procedure for creating a frailty index. BMC Geriatr 8:24. 10.1186/1471-2318-8-24 18826625 10.1186/1471-2318-8-24 PMC2573877

[CR42] Segerstrom SC, Miller GE (2004) Psychological stress and the human immune system: a meta-analytic study of 30 years of inquiry. Psychol Bull 130(4):601–630. 10.1037/0033-2909.130.4.601 15250815 10.1037/0033-2909.130.4.601 PMC1361287

[CR43] Sommerlad A, Ruegger J, Singh-Manoux A, Lewis G, Livingston G (2018) Marriage and risk of dementia: systematic review and meta-analysis of observational studies. J Neurol Neurosurg Psychiatry 89(3):231–238. 10.1136/jnnp-2017-316274 29183957 10.1136/jnnp-2017-316274 PMC5869449

[CR44] Sundström A, Westerlund O, Kotyrlo E (2016) Marital status and risk of dementia: a nationwide population-based prospective study from Sweden. BMJ Open 6(1):e008565. 10.1136/bmjopen-2015-008565 26729377 10.1136/bmjopen-2015-008565 PMC4716184

[CR45] Trevisan C, Veronese N, Maggi S, Baggio G, De Rui M, Bolzetta F, Zambon S, Sartori L, Perissinotto E, Crepaldi G, Manzato E, Sergi G (2016) Marital status and frailty in older people: gender differences in the Progetto Veneto Anziani longitudinal study. J Womens Health (Larchmt) 25(6):630–637. 10.1089/jwh.2015.5592 26845424 10.1089/jwh.2015.5592

[CR46] Trevisan C, Veronese N, Maggi S, Baggio G, Toffanello ED, Zambon S, Sartori L, Musacchio E, Perissinotto E, Crepaldi G, Manzato E, Sergi G (2017) Factors influencing transitions between frailty states in elderly adults: the Progetto Veneto Anziani longitudinal study. J Am Geriatr Soc 65(1):179–184. 10.1111/jgs.14515 27861714 10.1111/jgs.14515

[CR47] Trevisan C, Grande G, Vetrano DL, Maggi S, Sergi G, Welmer AK, Rizzuto D (2020) Gender differences in the relationship between marital status and the development of frailty: a Swedish longitudinal population-based study. J Womens Health (Larchmt) 29(7):927–936. 10.1089/jwh.2019.8095 32298606 10.1089/jwh.2019.8095

[CR48] Vidarsdottir H, Fang F, Chang M, Aspelund T, Fall K, Jonsdottir MK, Jonsson PV, Cotch MF, Harris TB, Launer LJ, Gudnason V, Valdimarsdottir U (2014) Spousal loss and cognitive function in later life: a 25-year follow-up in the AGES-Reykjavik study. Am J Epidemiol 179(6):674–683. 10.1093/aje/kwt321 24444551 10.1093/aje/kwt321 PMC3939848

[CR49] Wang Y, Karlsson R, Lampa E, Zhang Q, Hedman AK, Almgren M, Almqvist C, McRae AF, Marioni RE, Ingelsson E, Visscher PM, Deary IJ, Lind L, Morris T, Beck S, Pedersen NL, Hagg S (2018) Epigenetic influences on aging: a longitudinal genome-wide methylation study in old Swedish twins. Epigenetics 13(9):975–987. 10.1080/15592294.2018.1526028 30264654 10.1080/15592294.2018.1526028 PMC6284777

[CR50] Whisman MA, Robustelli BL, Sbarra DA (2016) Marital disruption is associated with shorter salivary telomere length in a probability sample of older adults. Soc Sci Med 157:60–67. 10.1016/j.socscimed.2016.03.029 27062452 10.1016/j.socscimed.2016.03.029 PMC4883574

[CR51] Wolf EJ, Maniates H, Nugent N, Maihofer AX, Armstrong D, Ratanatharathorn A, Ashley-Koch AE, Garrett M, Kimbrel NA, Lori A, Va Mid-Atlantic Mirecc W, Aiello AE, Baker DG, Beckham JC, Boks MP, Galea S, Geuze E, Hauser MA, Kessler RC, Koenen KC, Miller MW, Ressler KJ, Risbrough V, Rutten BPF, Stein MB, Ursano RJ, Vermetten E, Vinkers CH, Uddin M, Smith AK, Nievergelt CM, Logue MW (2018) Traumatic stress and accelerated DNA methylation age: a meta-analysis. Psychoneuroendocrinology 92:123–134. 10.1016/j.psyneuen.2017.12.007 29452766 10.1016/j.psyneuen.2017.12.007 PMC5924645

[CR52] Yen YC, Lung FW (2013) Older adults with higher income or marriage have longer telomeres. Age Ageing 42(2):234–239. 10.1093/ageing/afs122 22951603 10.1093/ageing/afs122 PMC3575119

[CR53] Ying K, Liu H, Tarkhov AE, Sadler MC, Lu AT, Moqri M, Horvath S, Kutalik Z, Shen X, Gladyshev VN (2024) Causality-enriched epigenetic age uncouples damage and adaptation. Nature Aging 4(2):231–246. 10.1038/s43587-023-00557-0 38243142 10.1038/s43587-023-00557-0 PMC11070280

[CR54] Young AC, Glaser K, Spector TD, Steves CJ (2016) The identification of hereditary and environmental determinants of frailty in a cohort of UK twins. Twin Res Hum Genet 19(6):600–609. 10.1017/thg.2016.72 27719687 10.1017/thg.2016.72

[CR55] Yu YL, Liu H (2020) Marital quality and salivary telomere length among older men and women in the United States. J Aging Health. 10.1177/0898264320980250 33371776 10.1177/0898264320980250 PMC8122040

[CR56] Yu R, Wong M, Chong KC, Chang B, Lum CM, Auyeung TW, Lee J, Lee R, Woo J (2018) Trajectories of frailty among Chinese older people in Hong Kong between 2001 and 2012: an age-period-cohort analysis. Age Ageing 47(2):254–261. 10.1093/ageing/afx170 29161361 10.1093/ageing/afx170

[CR57] Zannas AS, Arloth J, Carrillo-Roa T, Iurato S, Röh S, Ressler KJ, Nemeroff CB, Smith AK, Bradley B, Heim C, Menke A, Lange JF, Brückl T, Ising M, Wray NR, Erhardt A, Binder EB, Mehta D (2015) Lifetime stress accelerates epigenetic aging in an urban, African American cohort: relevance of glucocorticoid signaling. Genome Biol 16:266. 10.1186/s13059-015-0828-5 26673150 10.1186/s13059-015-0828-5 PMC4699359

[CR58] Zhang YR, Xu W, Zhang W, Wang HF, Ou YN, Qu Y, Shen XN, Chen SD, Wu KM, Zhao QH, Zhang HN, Sun L, Dong Q, Tan L, Feng L, Zhang C, Evangelou E, Smith AD, Yu JT (2022) Modifiable risk factors for incident dementia and cognitive impairment: an umbrella review of evidence. J Affect Disord 314:160–167. 10.1016/j.jad.2022.07.008 35863541 10.1016/j.jad.2022.07.008

