# Supplementary material for: Marital and living status and biological ageing trajectories: a longitudinal cohort study with a 20-year follow-up
Source: Biogerontology. 2025 Jan 8;26(1):34. doi: 10.1007/s10522-024-10171-1 (PMC11711563; doi:10.1007/s10522-024-10171-1)
Supplement: Supplementary file 1 — Supplementary file1 (PDF 363 KB) [file 10522_2024_10171_MOESM1_ESM.pdf]

Supplementary Tables and Figures

List of contents

eTables

eTable 1 The construction of BAs

eTable 2 The 42 items included in the Rockwood-based FI

eTable 3 The association between marital and living status and the baseline BA measurement among people with constant marital and living status

eTable 4 The association between baseline marital and living status and repeated BA measurements by sex

eFigures

eFigure 1 The SATSA timeline

eFigure 2 Transformation of FI

eTables

eTable 1 The construction of BAs

	Component elements	Measurement	Statistical methods
TL (17)	Leukocyte telomere length	qPCR	Ratio of measured telomere length to a reference length
Horvath (6) DNAmAge	Leukocyte DNA Methylation on 353 age-associated CpGs	Infinium HumanMethylation 450K BeadChip	Elastic net regression (regressing CA on CpGs)
FI (21)	42 health deficits	Self-reported questionnaire	Ratio of the number of deficit presented to the total number of deficit considered
Cognitive function (8)	Verbal ability, spatial ability, memory, and perceptual speed	In-person cognitive testing	Principal component analysis

Note: BA, biological age; DNAmAge, DNA methylation age estimator; TL: telomere length; FI, frailty index; qPCR, quantitative polymerase chain reaction; CpG, cytosine nucleotide being followed by a guanine nucleotide.

eTable 2 The 42 items included in the Rockwood-based FI

Item	Scoring
Hearing status	Perfect=0, Good=0.25, Pretty Good=0.5, Bad=0.75, Deaf or almost deaf=1
Vision status	Perfect=0, Good=0.25, Pretty Good=0.5, Bad=0.75, Blind or almost blind=1
Health prevents from doing things normally would like to do	No=0, Somewhat=0.5, Yes=1
Self-reported general health	Good=0, Mediocre=0.5, Bad=1
Cancer or leukemia	No=0, Yes=1
Rheumatoid arthritis	No=0, Yes=1
Arthritis	No=0, Yes=1
Chronic bronchitis or emphysema	No=0, Yes=1
Cataracts	No=0, Yes=1
Chest pain	No=0, Yes=1
Circulation problems in arms or legs	No=0, Yes=1
Persistent cough	No=0, Yes=1
Diabetes	No=0, Yes=1
Goiter or other gland problems	No=0, Yes=1
Heart failure	No=0, Yes=1
Hypertension	No=0, Yes=1
Kidney disease	No=0, Yes=1
Brittle bones	No=0, Yes=1
Sciatica	No=0, Yes=1
Anemia	No=0, Yes=1
Cerebral hemorrhage or blood clot in brain	No=0, Yes=1
Dizziness	No=0, Yes=1
Gastric ulcer	No=0, Yes=1
Allergies/allergic manifestations	No=0, Yes=1
Asthma	No=0, Yes=1
Shower and bathe [a]	No problem=0, Needs help=0.5, Cannot=1
Get in and out of bed [a]	No problem=0, Needs help=0.5, Cannot=1
Dress and undress [a]	No problem=0, Needs help=0.5, Cannot=1
Self-grooming [a]	No problem=0, Needs help=0.5, Cannot=1
Walking [a]	No problem=0, Needs help=0.5, Cannot=1
Trouble getting to toilet in time [a]	No=0, Yes=1
Travel further distances [b]	Can travel alone=0, Can go by taxi=0.5, Needs helper, special assistance or doesn't travel=1
Housework [b]	No problems=0, Needs help=0.5, Doesn't do=1
Prepare meals [b]	Can plan/prepare=0, Can heat up=0.5, Doesn't cook=1
Manage medications [b]	No problems=0, Needs help=0.5, Doesn't do=1
Manage money [b]	No problems=0, Needs help=0.5, Doesn't do=1
Use telephone [b]	Can look up numbers and dial=0, Needs help or doesn't use phone=1
Grocery shopping [b]	Can shop=0, Needs help=0.5, Doesn't shop=1
Feeling lonely [c]	Never, almost never, rather seldom=0, Quite often, always, almost always=1
Feeling depressed [c]	Never, almost never or rather seldom=0, Quite often, always, almost always=1
Consider oneself happy and carefree	No=1, Yes=0
Usually feels tired	No=0, Yes=1

Note: [a] from the instrument of Basic Activities of Daily Living, [b] from the instrument of Instrumental Activities of Daily Living, [c] from the Center for Epidemiologic Studies Depression Scale

eTable 3 The association between marital and living status and the baseline BA measurement among people with constant marital and living status

		Model 1	Model 2
**BA measurements**	Number of participants	β and 95% CI	β and 95% CI
**Telomere length (T/S ratio)**			
Marital status	404		
Married/cohabiting	275	Reference	Reference
Unmarried/non-cohabiting	129	-0.005 (-0.266, 0.255)	-0.002 (-0.267, 0.260)
Living status	384		
Living with someone	292	Reference	Reference
Living alone	92	-0.079 (-0.409, 0.251)	-0.060 (-0.392, 0.271)
**DNAmAge Horvath (years)**			
Marital status	338		
Married/cohabiting	237	Reference	Reference
Unmarried/non-cohabiting	101	0.141 (-0.061, 0.342)	0.144 (-0.055, 0.341)
Living status	323		
Living with someone	253	Reference	Reference
Living alone	70	0.146 (-0.077, 0.368)	0.171 (-0.051, 0.390)
**Cognitive function**			
Marital status	549		
Married/cohabiting	382	Reference	Reference
Unmarried/non-cohabiting	167	-0.048 (-0.217, 0.120)	-0.028 (-0.187, 0.130)
Living status	523		
Living with someone	402	Reference	Reference
Living alone	121	0.091 (-0.100, 0.281)	0.097 (-0.083, 0.277)
**Frailty index (square root)**			
Marital status	1326		
Married/cohabiting	854	Reference	Reference
Unmarried/non-cohabiting	472	0.298 (0.184, 0.413)	0.285 (0.169, 0.399)
Living status	1283		
Living with someone	945	Reference	Reference
Living alone	338	0.192 (0.065, 0.319)	0.183 (0.056, 0.309)

Note: Mixed models were used to estimate the association between baseline marital status and the first BA measurement in one-SD increase, with fixed effects for chronological age and sex in Model 1, and additionally for educational attainment, smoking status, BMI and birth year in 10-year interval in Model 2, and with random intercepts at the twin-pair level.
BA: biological age; CI: confidence interval.

eTable 4 The association between baseline marital and living status and repeated BA measurements by sex

**BA measurements**	Number of participants	Total measurements	β and 95% CI	*P* of Interaction term
**Telomere length (T /S ratio)**				
**Male**				
Marital status				
Married/cohabiting	157	391	Reference	
Unmarried/non-cohabiting	34	65	-0.041 (-0.351, 0.266)	
**Female**				
Marital status				
Married/cohabiting	118	302	Reference	
Unmarried/non-cohabiting	95	210	0.069 (-0.156, 0.296)	0.574
**DNAmAge Horvath (years)**				
**Male**				
Marital status				
Married/cohabiting	129	332	Reference	
Unmarried/non-cohabiting	24	45	0.289 (-0.016, 0.588)	
**Female**				
Marital status				
Married/cohabiting	108	286	Reference	
Unmarried/non-cohabiting	77	176	0.016 (-0.177, 0.211)	0.137
**Cognitive function**				
**Male**				
Marital status				
Married/cohabiting	202	800	Reference	
Unmarried/non-cohabiting	41	132	-0.216 (-0.470, 0.038)	
**Female**				
Marital status				
Married/cohabiting	180	644	Reference	
Unmarried/non-cohabiting	126	401	-0.032 (-0.218, 0.153)	0.257
**Frailty index (square root)**				
**Male**				
Marital status				
Married/cohabiting	451	2242	Reference	
Unmarried/non-cohabiting	144	496	0.297 (0.136, 0.458)	
**Female**				
Marital status				
Married/cohabiting	403	2079	Reference	
Unmarried/non-cohabiting	328	1293	0.287 (0.156, 0.417)	0.921

Note: Mixed models were used to estimate the association between baseline marital status and repeated BA measurement in one-SD increase, with fixed effects for chronological age, educational attainment, smoking status, BMI, calendar year of birth in 10-year interval, sex, and an interaction term between marital status and sex, and with random intercepts at the twin-pair level and the individual level. The strata-specific effect is shown. BA: biological age; CI: confidence interval.

eFigures

eFigure 1 the SATSA timeline

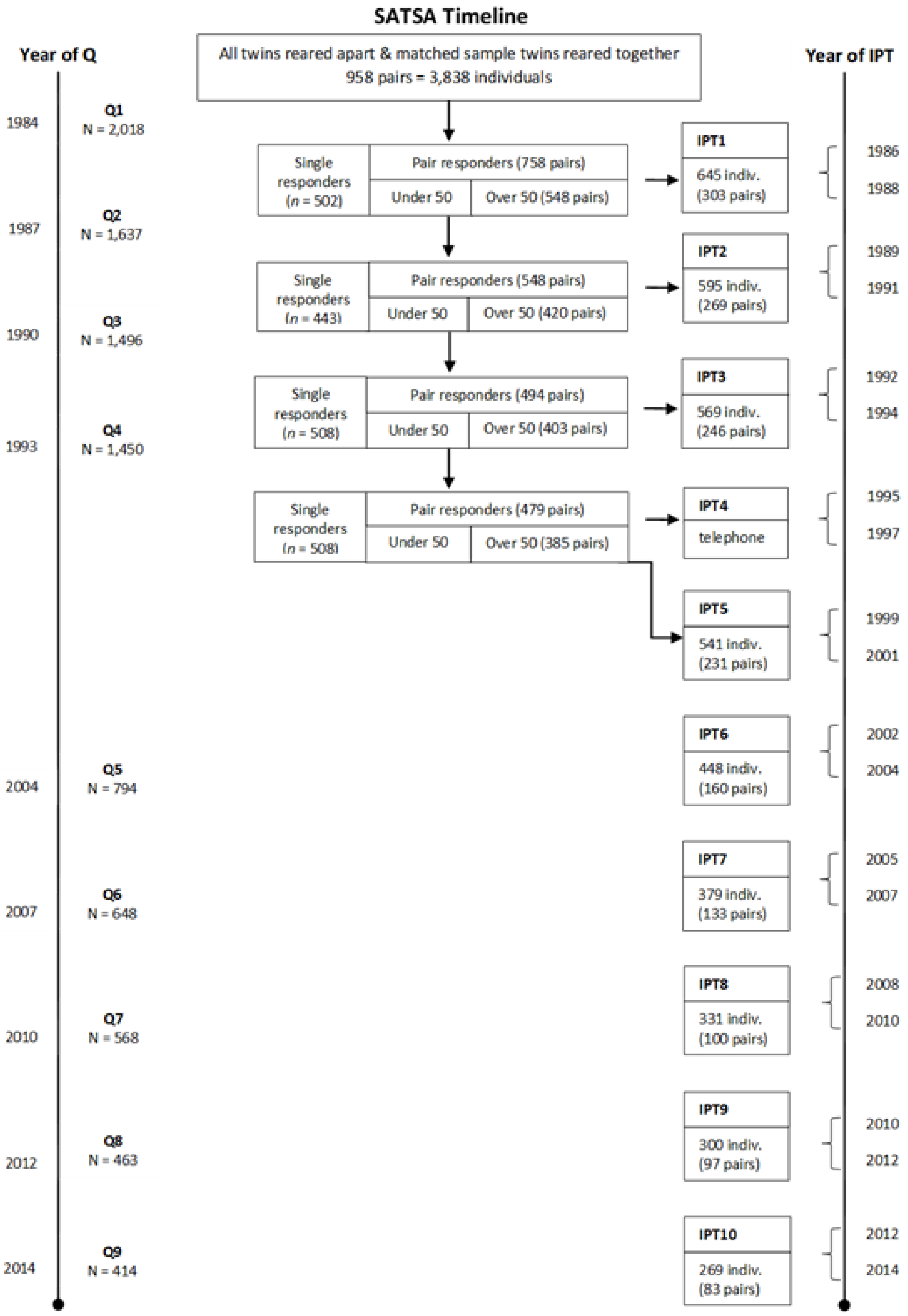

eFigure 2 Transformation of FI

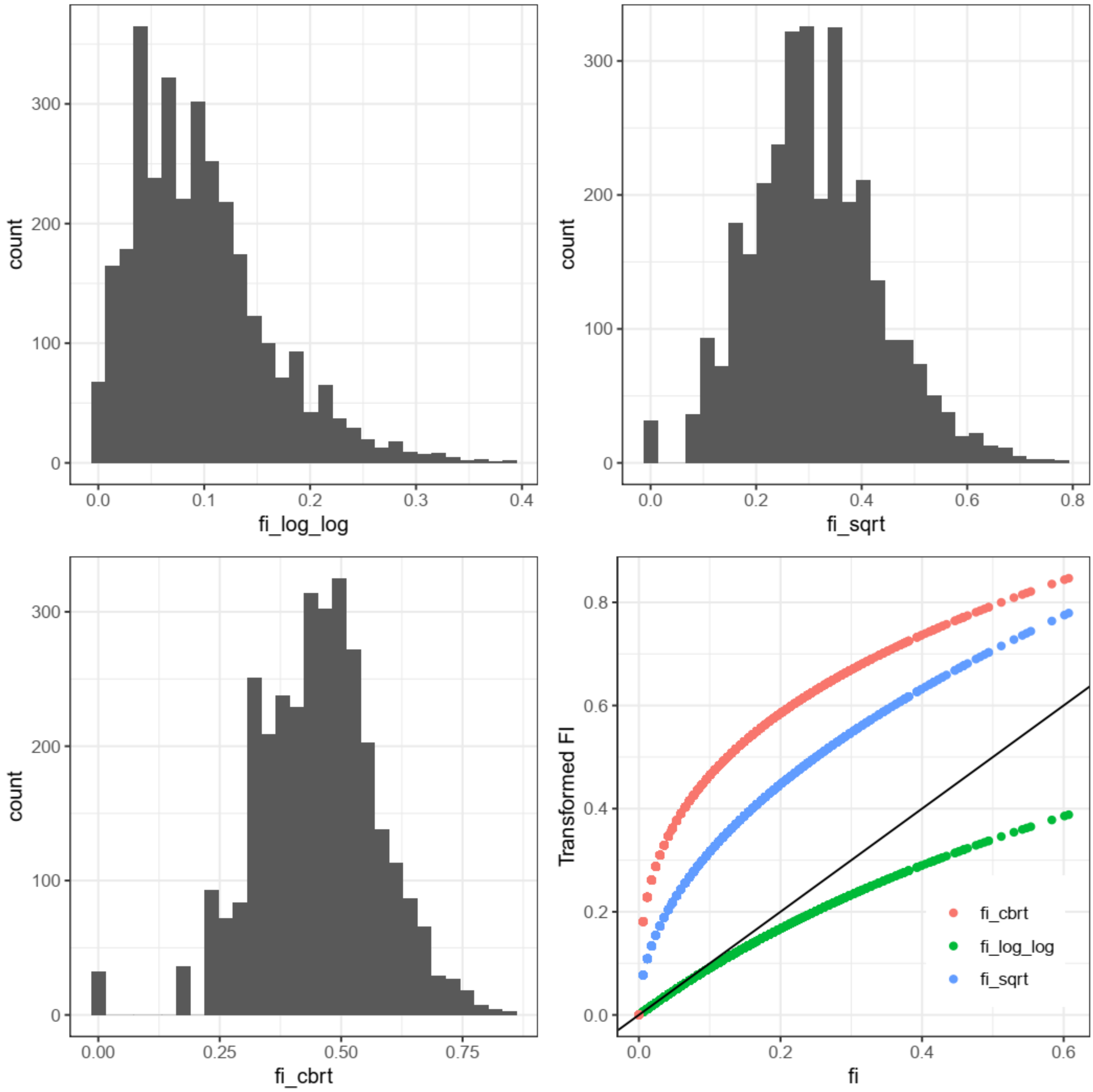

Due to the right skew of the FI distribution, we transformed data into square root (sqrt) of FI to better fit the normal distribution.
